# Examining the Potential Link Between Forkhead Box P1 and Severity and Social Impairment in Children with Autism Spectrum Disorder

**DOI:** 10.3390/jcm14207132

**Published:** 2025-10-10

**Authors:** Laila Yousef Al-Ayadhi, Nadra Elyass Elamin, Durria Ahmed Abdulmaged, Aurangzeb Taj Halepota, Dost Muhammad Halepoto

**Affiliations:** 1 Autism Research and Treatment Center, Department of Physiology, College of Medicine, King Saud University, P.O. Box 2925, Riyadh 11461, Saudi Arabia; ayadh2@gmail.com (L.Y.A.-A.); durahmed85@gmail.com (D.A.A.); dr_m_halepota@yahoo.com (D.M.H.); 2 Department of Physiology, College of Medicine, King Saud University, P.O. Box 2925, Riyadh 11461, Saudi Arabia; athalepota@yahoo.com

**Keywords:** autism spectrum disorder, Forkhead box protein P1, childhood autism rating scale, social responsiveness scale

## Abstract

**Background:** Autism spectrum disorder (ASD) is a complex neurodevelopmental condition characterized by impaired social skills and communication. Forkhead box protein P1 (FOXP1) is involved in the development of the brain and the pathogenesis of ASD. However, the function of FOXP1 within the brain remains unclear. The aim of this case–control study was to evaluate whether FOXP1 could be used as a diagnostic biomarker for ASD. **Method:** Blood plasma was collected from children with ASD and age-matched controls. The enzyme-linked immunosorbent assay (ELISA) was used to determine the FOXP1 plasma levels in ASD and control groups. The behavioral and social impairments in children with ASD were assessed using the Childhood Autism Rating Scale (CARS) and the Social Responsiveness Scale (SRS). Spearman’s correlation coefficient (r) was used to determine the correlation between different variables. **Results:** The plasma FOXP1 protein level was significantly decreased in children with ASD compared to the controls (*p* < 0.001). CARS showed significant differences between the mild-to-moderate and severe subgroups, while the SRS showed no significant difference between the two subgroups. Moreover, both the mild-to-moderate and severe subgroups exhibited a substantial drop in plasma FOXP1 compared to the controls. ASD children older than six years old also showed a significantly decreased FOXP1 level, compared to those aged six years or less. Furthermore, no significant correlation between the FOXP1 level, CARS, and SRS was observed. However, a negative correlation was found between age and FOXP1 plasma level. **Conclusions:** We suggest that plasma FOXP1 may act as a potential biomarker for the prognosis of ASD severity and social communication impairment. Further research with a larger sample size is needed to clarify these associations and help diagnose or understand the underlying mechanism of ASD.

## 1. Introduction

Autism spectrum disorder (ASD) is a complex neurodevelopmental condition characterized by the early onset of a wide range of symptoms and different levels of impairments, including social communication, language, and the presence of restricted and repetitive behaviors and interests. Individuals with ASD may present additional co-occurring conditions, such as intellectual disability (ID), epilepsy, and gastrointestinal (GI) dysfunction [1,2]. The etiology of ASD is multifactorial; it involves a combination of genetic and environmental factors, physiological and metabolic abnormalities including inflammation, immunity, and oxidative stress, as well as GI dysfunction [3]. Diagnosing ASD is challenging due to the complexity and heterogeneity of the symptoms. The diagnosis depends on behavioral and educational assessment to examine the cognitive ability of ASD subjects. Recently, neurodiversity and combined receiver operator characteristic (ROC) curve analysis approaches were introduced to improve the understanding and diagnosis of ASD [4,5]. Over the past two decades, a wide range of biological markers have been investigated to improve the diagnosis and characterization of ASD. Most biomarker research has focused on genetic, proteomic, immune, and metabolic indicators. For example, alterations in cytokine profiles, markers of oxidative stress, mitochondrial function, and neurotransmitter pathways have been reported in children with ASD, suggesting potential disruptions in neuroimmune and metabolic regulation [6,7,8]. Therefore, efforts have been made to identify blood biomarkers and target core symptoms to improve diagnostic accuracy, early diagnosis, and therapeutic strategies. Detection of blood biomarkers is favorable due to the feasibility, speed, and comparably low cost of this method compared to other techniques [3,9]. Commonly studied candidates, such as brain-derived neurotrophic factor (BDNF), serotonin, and various cytokines, have shown inconsistent results regarding their diagnostic utility [3,7,8].

Previous research has identified hundreds of genes that contribute to the etiopathology of ASD. These genes are associated with an increased risk of ASD development. The FOXP1 gene is considered one of the five high-risk ASD genes [10,11]. Within this context, FOXP1 has emerged as a particularly relevant candidate. Unlike many nonspecific metabolic or inflammatory markers, FOXP1 is directly implicated in molecular pathways underlying social communication and cognitive development, which are core domains affected in ASD.

Forkhead Box P1 (FOXP1) is a member of subfamily P of the forkhead box (FOX) transcription factor family [12]. It is widely expressed in the developing and adult brain, and it plays a key role during early neural development [13]. FOXP1 is involved in regulating different brain regions, brain function, and normal processes by interacting with FOXP2 and other transcriptional regulators. It plays a crucial role in the regulation of striatal development and function, hippocampal function, and neuro-glial communication [13,14,15]. The FOXP1 protein is widely expressed in various human tissues such as the brain, heart, and lungs, as well as immune system, and neurons and controls their developmental processes [16,17].

Mutation or haploinsufficiency of the FOXP1 gene has been implicated in FOXP1 syndrome, a neurodevelopmental disorder characterized by developmental delay, ID, speech and language impairment, different levels of cognitive abilities, and behavior abnormalities [18,19,20]. The syndrome also manifests characteristic symptoms of ASD [13,15,21,22,23,24].

Increasing research evidence suggests the link between FOXP1 and neurodevelopmental disorders (NDDs), such as ASD and ID [25]. Individuals with FOXP1 loss-of-function or mutation of the gene revealed high cognitive impairment [26]. FOXP1 is also associated with different medical problems such as cardiac, renal, and gastrointestinal conditions.

A growing body of research has investigated the contributing role of FOXP1 in ASD. Research on FOXP1 knock-out mouse models showed striatum developmental defects and autism-like behaviors in the brains of mice, including communication impairments, and decreased sociability [27,28]. In the brain FOXP1 loss-of-function results in social behavior deficit, as well as impaired learning, and memory [29]. Several studies on knock-out mouse models and individuals with ASD identified mutations and deletion in the FOXP1 gene in individuals with ASD. Furthermore, an association between the mutation of FOXP1 and increased gene expression in children with ASD and/or ID was reported [12,24]. Araujo et al., additionally reported an association of loss-of-function with ASD in human neural cells and FOXP1 knockout mice [14]. FOXP1 also contributes to the gastrointestinal dysfunction in ASD by altering the motility and achalasia [30]. On top of that, mutation of FOXP1 was found to induce cortical neuron development, causing ASD [31].

Forkhead Box P1 protein has been increasingly researched for its impact on ASD severity and social impairment. Bacon et al. demonstrated that FOXP1 deficiency is associated with various cognitive and social deficits, including hyperactivity, increased repetitive behavior, anxiety, and reduced social interests [28]. Previous studies reported that abnormal development in brain regions including the cerebral cortex, striatum, amygdala, and cerebellum, in addition to reduced excitatory synaptic transmission, and E/I imbalance are connected to autistic-like behaviors and social behavior impairment [2]. Disruption of FOXP1 affects the brain regions involved in social communication, inducing social dysfunction [14,32]. Recently, a study on FOXP1-cKO mice exhibited sensory deficits, hyper-reactivity and avoidance behavior, and social and communication difficulties in ASD [33]. Furthermore, Shiota et al. investigated the genetic variants of FOXP1 in Japanese children with ASD. They revealed the association of FOXP1 among other genes with social impairment [34].

To date, all studies that investigated the impact of FOXP1 in ASD have been genetic studies. Therefore, evaluating FOXP1 alongside or in comparison to previously studied biomarkers may provide additional insight into the biological basis of ASD and improve the specificity of biomarker-based approaches. In this study, we aimed to measure, for the first time, the plasma level of FOXP1 in autistic children, compared to healthy controls. We also assessed its correlation with disease severity and social and behavioral impairment, using Childhood Autism Rating Scale (CARS) [35] and Social Responsiveness Scale (SRS) [36].

## 2. Materials and Methods

### 2.1. Participants

Eighty children were enrolled in the case–control study, namely, 40 ASD children and 40 age-matched healthy children. ASD children (35 males and 5 females) aged 3 to 10 years [median (IQR), 6 (3)] were recruited from the Autism Research and Treatment Center (ARTC), College of Medicine, King Saud University. ASD children were diagnosed by a qualified clinician, using the Diagnostic and Statistical Manual of Mental Disorders, (DSM-V) [1]. A combination of medical history review, parental reports, and neuropsychiatric assessment were used to assess all participants for past or current medical illness. ASD children with associated neurological diseases (such as palsy and tuberous sclerosis), metabolic disorders (e.g., phenylketonuria, or diabetes), or any autoimmune disease were excluded from the study. The control group consisted of 40 healthy children (33 males and 7 females) aged 3 to 12 years [median (IQR), 6 (6)] who were enrolled from the pediatric clinic at King Khalid University Hospital. They did not have any clinical signs of immunological, chronic, or neuropsychiatric diseases.

The study was approved by the Institutional Review Board, College of Medicine, King Saud University, Riyadh, Saudi Arabia, according to the most recent Declaration of Helsinki. Informed consent was obtained from the parents or legal guardians of all study subjects before participation.

### 2.2. Blood Samples

Blood samples (5 mL) were obtained from all participants after overnight fasting. Blood was centrifuged at 3500 rpm at 4 °C for 15 min. Then plasma was collected, aliquoted, and stored at −80 °C until analysis.

### 2.3. Behavioral Assessment of ASD Children

All behavioral assessments were conducted by trained raters who were blinded to the participants’ diagnosis during the assessment process to minimize potential bias. The degree of ASD severity was assessed using the Childhood Autism Rating Scale (CARS) which rates the child in 15 areas. A score of 30 to 36 points on this scale indicates mild-to-moderate autism, and a score of 37 to 60 points indicates severe autism [35].

The Social Responsiveness Scale (SRS) was used to identify the presence and severity of social impairment. It is a quantitative parent/interviewer-report measure of autistic behaviors; it measures five domains of social deficits: social awareness, social cognition, social communication, social motivation, and autistic mannerisms. A total score of 60–75 is considered to indicate mild-to-moderate range of social impairment, while a total score of 76 or higher on the SRS is considered to represent severe social impairment [36].

### 2.4. Assessment of Forkhead Box P1 (FOXP1) Plasma Levels

The plasma levels of FOXP1 were evaluated using commercially available sandwich ELISA kits (Cusabio Biotech Co. Ltd., Wuhan, China, Cat# E15456h), according to the manufacturer’s instruction. Briefly, a microtiter plate was pre-coated with an antibody specific for FOXP1. Standards or samples were then added to the appropriate microtiter plate wells with a biotin-conjugated polyclonal antibody preparation specific for FOXP1. Next, avidin conjugated to horseradish peroxidase (HRP) was added to each microplate well and incubated for two hours at 37 °C. Then, a 3,3′, 5,5′ tetramethylbenzidine (TMB) substrate solution was added to each well. Only those wells that contained FOXP1, biotin-conjugated antibody and enzyme-conjugated avidin exhibited a change in color. The reaction was terminated via the addition of a sulfuric acid solution and the color change was measured spectrophotometrically at a wavelength of 450 nm. The concentration of FOXP1 in the samples was then determined by comparing the optical density of the samples to the standard curve. Plasma FOXP1 levels were measured in duplicate for each sample to avoid inter-assay variation.

### 2.5. Statistical Analysis

The results were analyzed using Statistical Package for the Social Science software, version 26 (SPSS) (IBM Corp., Chicago, IL, USA). All data were tested for normality using the Shapiro–Wilk test. The data were non-parametric, and are presented as the median and interquartile range (median (IQR). The Mann–Whitney test was used to compare the variables of the studied groups. Spearman’s correlation coefficient ‘r’ was used to determine the relationship between different variables. *p* < 0.05 was considered significant for all tests.

## 3. Results

**Figure 1:**
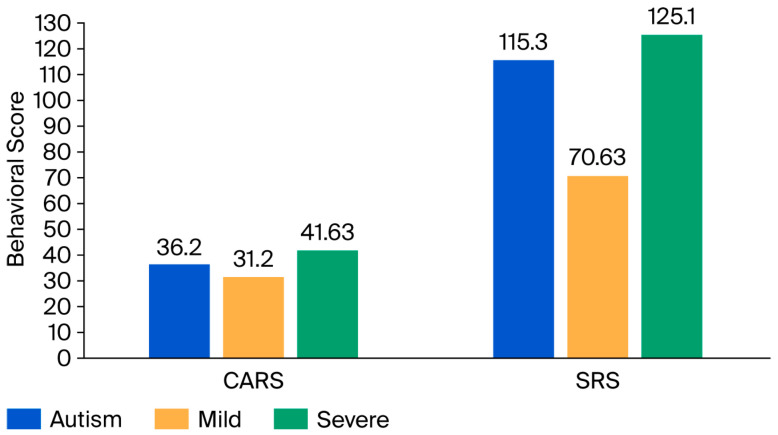
Mean behavioral (CARS, and SRS) scores in children with ASD and their association with ASD severity.

Table 1 shows the general characteristics, and plasma levels of FOXP1 in ASD and control groups and their association with the CARS, SRS, and age. Data are presented as median (IQR). The plasma FOXP1 level was significantly decreased in the ASD group compared to the control group (*p* < 0.05). Furthermore, it was noted that an almost 67% decrease in FOXP1 level was recorded in children with severe ASD compared to the control group. In the ASD group, the association between the FOXP1 level and the severity of ASD and social behavior impairment was measured using the CARS and SRS. ASD subjects were subcategorized into mild-to-moderate and severe subgroups according to their score (Figure 1).

The CARS analysis showed that 52.5% of the ASD patients (n = 21) had mild-to-moderate autism (≤36.5), while 47.5% of children (n = 19) had severe autism (˃36.5). There was a significant decrease in plasma FOXP1 levels in the severe subgroup of ASD compared to the mild-to-moderate ASD subgroup (*p* ≤ 0.05), suggesting that the decreased level of FOXP1 is associated with the severity of ASD. Also, there was a significant decrease in plasma levels of FOXP1 in both mild-to-moderate and severe ASD subgroups compared to the control group (*p* = 0.045, *p* ≤ 0.01, respectively).

**Figure 2:**
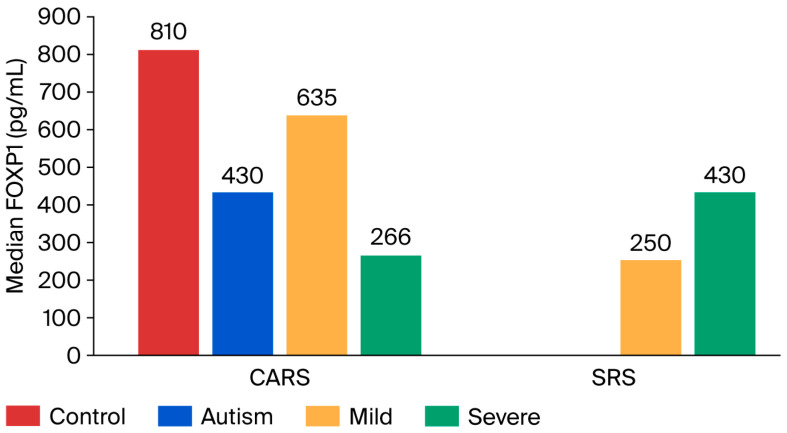
Median FOXP1 (pg/mL) levels in ASD and control groups.

Furthermore, SRS analysis showed that 20% of the ASD patients had moderate autism (score < 76), while 80% had severe autism (score ˃ 76). The SRS severe subgroup exhibited a remarkably higher median plasma FOXP1 level compared to the mild-to-moderate subgroup, but the difference did not reach any significant level (*p* ≥ 0.05). However, both the mild-to-moderate and severe subgroups exhibited a substantial drop in plasma FOXP1 compared to the controls (*p* = 0.016, *p* ≤ 0.01, respectively), suggesting the possible association with social impairment (Figure 2). Furthermore, a significantly lower level was observed in the ASD group children older than six years, compared to those aged six years or less (*p* = 0.037), as shown in Table 1.

**Figure 3:**
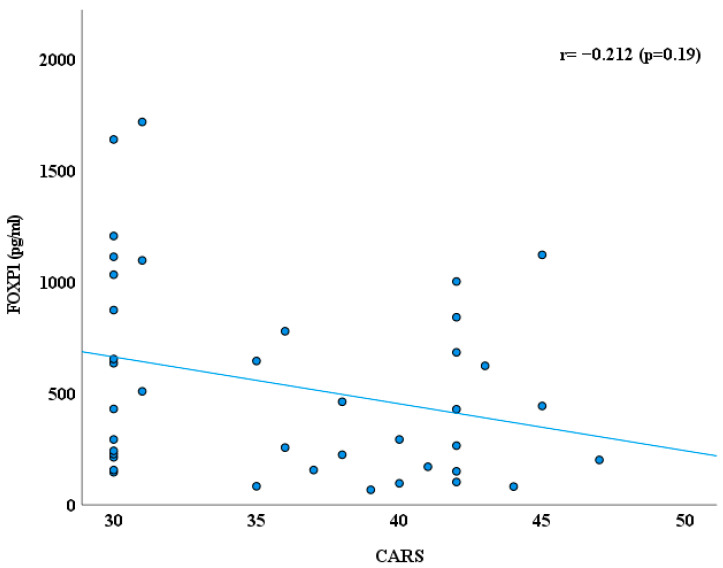
Correlation between FOXP1 protein (pg/mL) and CARS in ASD group.

**Figure 4:**
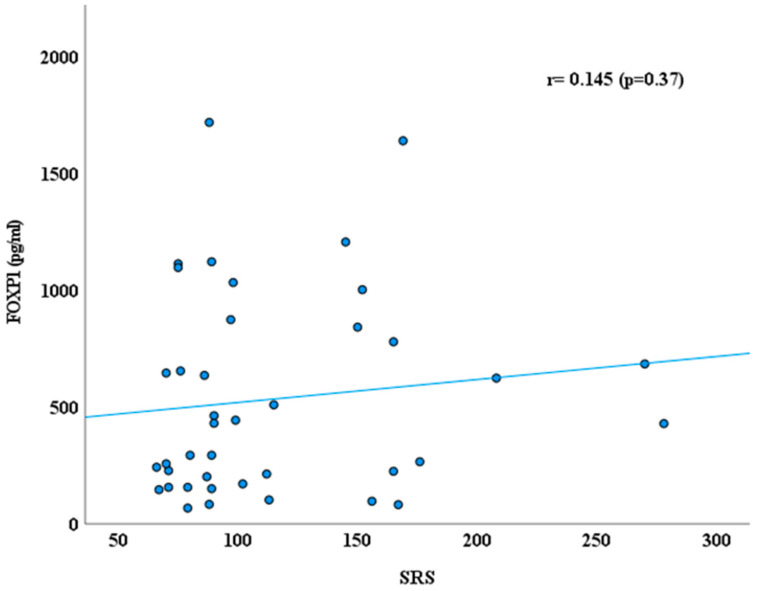
Correlation between FOXP1 protein (pg/mL) and SRS in ASD group.

**Figure 5:**
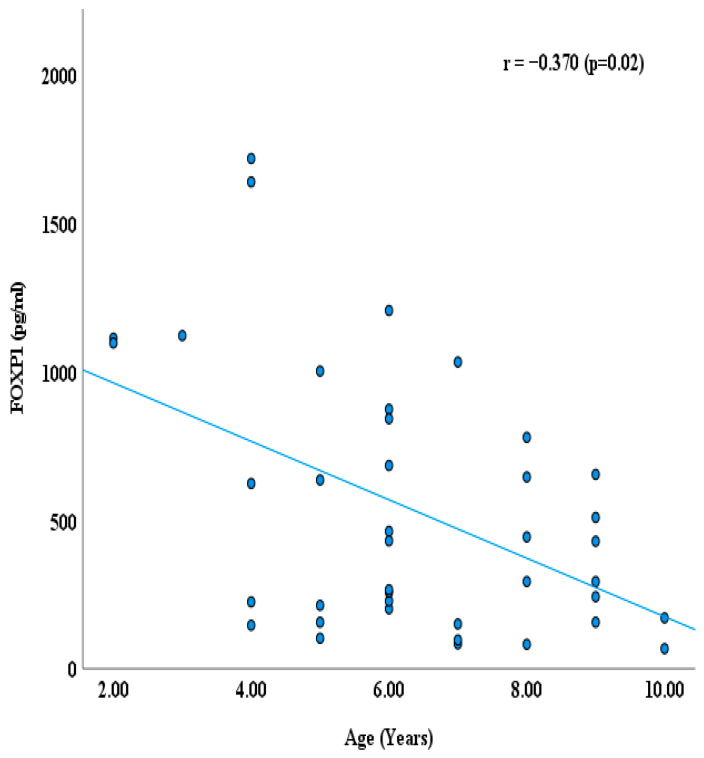
Correlation between FOXP1 protein (pg/mL) and age in ASD group.

Additionally, we explored the correlation between FOXP1 level, CARS, SRS, and age (Figure 3, Figure 4 and Figure 5) using Spearman correlation (r). Plasma FOXP1 exhibited no significant correlation with the CARS (r = −0.212; *p* = 0.19) and SRS (0.145, *p* = 0.37). However, a negative correlation between FOXP1 and age was observed (r = −0.369, *p* = 0.019).

## 4. Discussion

Impaired cognitive plasticity, characterized by restricted interests and repetitive behavior, is linked with unusual memory presentation in ASD, supporting hippocampal dysfunction. The dysregulated mitochondrial network and the resulting oxidative stress in the hippocampus contribute to impaired learning and cognitive impairment. FOXP1 may play a major role in similar alterations in ASD subjects. However, the function of FOXP1 within the brain remains mainly unclear [23,37].

Studies have identified mutations and deletions in the FOXP1 gene in individuals with ASD and a stronger association between specific types of FOXP1 mutations and ASD compared to ID has been suggested. Mouse models or individuals with FOXP1 exhibit impaired neuronal development and behavior-related ASD, such as repetitive behaviors and social interaction deficits [20,28,38]. Recently, individuals with FOXP1 loss-of-function or mutation of the gene revealed high cognitive impairment, which is associated with mitochondrial dysfunction and oxidative stress [23,26], GI dysfunction [30], severe language impairment and brain development dysfunction [24]. Given that all these impairments are hallmarks of ASD, we aimed to measure FOXP1 levels in the blood plasma of children with ASD, which may provide a possible disease severity biomarker and lead to novel medical treatments.

This study is the first to investigate the plasma level of FOXP1 protein in ASD children compared to controls, and its relationship with behaviors and social impairments in ASD children. The results of the present study showed significantly decreased FOXP1 plasma levels in children with ASD compared to the control group. According to the CARS, a measure of disease severity, the plasma FOXP1 levels were significantly different between the mild-to-moderate and severe subgroups. Moreover, the difference between the mild-to-moderate and severe subgroups was significantly decreased, compared to the control group, suggesting a possible role of FOXP1 in the pathophysiology of ASD. This finding should be interpreted with caution, as we were not able to find comparable data in the literature to compare our findings. However, our results are consistent with a recent study that demonstrated reduced gene expression or deficient activity of FOXP1 associated with ASD [23]. On the other hand, some previous studies suggested increased expression of FOXP1 in ASD subjects compared to the controls [12]. Research also showed that FOXP1 and FOXP2 are highly expressed in neurons of the developing cortex and striatum [25,39].

Any abnormalities during cortical development may result in the development of neurodevelopmental disorders including ASD [40]. Moreover, mutation leads to the disruption of normal neuronal development, affecting brain function, and connectivity [21,39]. In light of this, the decreased level of FOXP1 in the current study may affect cortical development or disrupt normal neuronal development in a subgroup of children with ASD. As the FOXP1 protein exerts an important role in maintaining normal network integrity for brain development, we speculate that a low plasma FOXP1 level may disrupt this network, leading to brain development abnormalities in regions that are responsible for cognitive and social interaction.

Furthermore, in the ASD group, a significantly lower FOXP1 level was observed in children older than six years compared to those aged six years or less (*p* = 0.013). Interestingly, FOXP1 levels exhibited a negative relationship with age (r = −0.0369, *p* = 0.02), suggesting that expression of the FOXP1 may be influenced by neurodevelopmental processes. This observation aligns with the possibility that FOXP1 is dynamically regulated across childhood and adolescence, potentially contributing to age-dependent differences in ASD pathophysiology. Future longitudinal studies are needed to clarify whether this age-related decline is specific to ASD or part of a broader developmental trajectory.

Since social dysfunction is the core symptom of ASD, the loss of FOXP1 function results in social behavior deficits, and impaired learning and memory [27,28,29]. In the current study, we found that 80% of ASD patients fell into the severe SRS range. The SRS severe subgroup exhibited a remarkably higher plasma FOXP1 level compared to the mild-to-moderate subgroup, but the difference did not reach any significant level (*p* > 0.05). This could be attributed to the great variability in the plasma levels. However, there was a substantial decrease in plasma levels of FOXP1 in both the mild-to-moderate and severe ASD subgroups compared to the control group (*p* = 0.016, *p* < 0.001, respectively), suggesting a possible association of social communication impairment observed in ASD children. Although the imbalance in SRS subgroups might limit the power of direct comparison, the overall results provide valuable insights into the patterns of symptom severity. This result should be interpreted with caution and required further validation in a larger and balanced cohort.

In our cohort, FOXP1 levels did not show significant correlations with behavioral severity as measured with the CARS and SRS. This lack of association suggests that while FOXP1 may reflect underlying neurobiological alterations in ASD, it may not directly track with clinical symptom severity. The observed trend may be driven by other factors, including the heterogeneity of ASD phenotype, age, and sample size. This finding raises important considerations regarding the specificity and clinical utility of FOXP1 as a biomarker. Rather than serving as a direct indicator of behavioral outcomes, FOXP1 may represent a biological signature of ASD risk or diagnosis, with its role in prognosis or symptom monitoring requiring further investigation.

In neurodevelopmental diseases [28,41], we may conclude that lower plasma concentrations of FOXP1 may contribute to impaired neuronal development in the brain of ASD [40] given the critical role of FOXP1 in the evolution of the human cerebral cortex and hippocampus. This study also provides evidence that higher FOXP1 may protect normal controls as compared to ASD subjects. It also supports the link between low FOXP1 levels in ASD and a potential association with neurodevelopmental changes.

## 5. Limitations

Our findings should be interpreted in light of certain limitations, including the relatively small sample size and the cross-sectional design. Additionally, children with overlapping symptoms were not excluded from the healthy controls, such as children with high degrees of anxiety, depression, attention deficit hyperactivity disorder (ADHD), or learning disorders. Another limitation that was not assessed is whether psychotropic medications or other treatments in the ASD group could be driving the changes in the biomarkers. These factors limit our ability to infer causality or assess developmental trajectories of FOXP1 expression. Larger, longitudinal studies will be essential validating FOXP1 as a candidate biomarker and determining its potential value in early diagnosis, prognosis, or stratification of ASD subtypes. If confirmed, FOXP1 could contribute to the development of more personalized interventions for children with ASD.

## 6. Conclusions

Plasma FOXP1 levels decreased significantly in individuals with ASD and negatively correlated with age. They also significantly decreased in the mild-to-moderate subgroup, suggesting the association of FOXP1 with the severity of the disease. FOXP1 shows promise as a potential biomarker for the prognosis of the severity of ASD and social communication impairment. The non-significant correlation between FOXP1 and symptom severity suggests that the observed trend may be driven by other factors, including the heterogeneity of ASD phenotype, age, and sample size. Therefore, further research with a larger sample size is needed to clarify these associations and improve the diagnosis and understanding of the underlying mechanism of ASD.

## Figures and Tables

**Table 1:** General characteristics, and plasma levels of FOXP1 in ASD and control groups, and their association with CARS, SRS, and age.

Variables	Group	n (Age in Years)	FOXP1 (pg/mL) Median (IQR)	Change	*p*-Value	Score
	Controls	40 (3–10)	810 (671–1102)	-	<0.01 *	-
CARS	Autism	40 (3–12)	430 (178–826)	47%	-	-
	Severe	19	266 (150–624)	67%	<0.01 ***	˃36.5
	Mild-to-moderate	21	635 (235–1065)	21%	0.045 ** 0.05 #	<36.5
SRS	Severe	32	430 (171–779)	47%	<0.01 ***	˃76
	Mild-to-moderate	8	250 (174–984)	69%	0.016 ** 0.83 #	<76
Age	˃6 years	17	293 (123–577)		0.04 §	-
	≤6 years	23	624 (225–1097)			-

* Comparing ASD children with control subjects; ** comparing mild-to-moderate with control subjects; *** comparing severe with control subjects; # comparing mild-to-moderate with severe subjects; § comparing the age of autistic subjects (*p*-value ≤ 0.05 was considered statistically significant).

## Data Availability

All data generated or analyzed during this study are available from the corresponding author upon request.
